# Public knowledge, attitudes, and barriers to cancer screening in the UAE: a comprehensive assessment to inform screening practices

**DOI:** 10.3389/fpubh.2026.1808218

**Published:** 2026-04-07

**Authors:** Anan S. Jarab, Walid Al-Qerem, Hamza Jarab, Omar Jrab, Rahma Elsherif, Ahmad Z. Al Meslamani, Zelal Kharaba, Yazid Al Hamarneh, Maher Khdour, Nada A. Alsaleh, Salahdein Aburuz

**Affiliations:** 1 Department of Clinical Pharmacy, Faculty of Pharmacy, Jordan University of Science and Technology, Irbid, Jordan; 2 Department of Pharmacy, Faculty of Pharmacy, Al-Zaytoonah University of Jordan, Amman, Jordan; 3 Faculty of Medicine, Jordan University of Science and Technology, Irbid, Jordan; 4 Barts and The London School of Medicine and Dentistry, Queen Mary University of London, Victoria, Malta; 5 College of Pharmacy, Al Ain University, Abu Dhabi, United Arab Emirates; 6 Department of Pharmacy Practice and Pharmacotherapeutics, College of Pharmacy, University of Sharjah, Sharjah, United Arab Emirates; 7 Department of Pharmacology, Faculty of Medicine and Dentistry, University of Alberta, Edmonton, AB, Canada; 8 Faculty of Pharmacy, Al-Quds University, Jerusalem, Palestine; 9 Department of Pharmacy Practice, College of Pharmacy, Princess Nourah Bint Abdulrahman University, Riyadh, Saudi Arabia; 10 Department of Pharmacology and Therapeutics, College of Medicine and Health Sciences, United Arab Emirates University, Al Ain, United Arab Emirates

**Keywords:** attitudes, barriers, cancer, knowledge, motivators, public, screening practices

## Abstract

**Background and objectives:**

Despite the proven benefits of early cancer detection in reducing disease-related complications and deaths, participation in cancer screening programs varies across population and is influenced by multiple individual and systemic-level factors. Understanding knowledge, attitudes and practices regarding cancer screening is essential to inform effective public health strategies, Therefore, this study aimed to assess these factors among adults in the UAE.

**Methods:**

A cross-sectional study was conducted using a bilingual (English/Arabic), self-administered online questionnaire distributed via social media. The survey collected data on socio-demographic characteristics, lifestyle-related risk factors, and cancer screening practices. Cancer knowledge was assessed using 59 items covering general knowledge, screenable cancers, warning signs and symptoms, and environmental risk factors. Attitudes perceived facilitators and barriers to screening were measured using a 5-point Likert scale. Screening behavior was assessed as self-reported lifetime (“ever”) participation in any cancer screening test. Binary logistic regression analysis was performed to identify factors associated with screening practices.

**Results:**

A total of 811 individuals completed the study questionnaire, of whom only 36.5% reported having ever undergone any cancer screening test. Higher knowledge (OR = 1.022; 95% CI: 1.007–1.037) and more positive attitudes (OR = 1.091; 95% CI: 1.062–1.120) were associated with increased screening uptake. Older age (OR = 0.970; 95% CI: 0.955–0.985) and lower income (OR = 0.689; 95% CI: 0.483–0.982) were associated with a reduced likelihood of screening. Physician recommendation (47.0%) and early detection (45.3%) were the main motivators, while fear of results (39.5%), lack of symptoms (38.6%), and unfamiliarity with screening sites (38.5%) were the most common barriers.

**Conclusion:**

The findings demonstrate moderate cancer knowledge, variable screening attitudes, and multiple behavioral and system-level barriers. Knowledge, attitudes, age, and income, were significantly associated with lifetime screening participation, while physician recommendation and early detection emerged as key motivators. Targeted educational interventions and improved healthcare system navigation may enhance screening uptake in similar populations. However, findings should be interpreted with caution due to cross-sectional design, convenience sampling, reliance on self-reported lifetime screening, and the relatively young age distribution, which may limit causal inference and generalizability.

## 1 Introduction

Cancer is a group of diseases characterized by the uncontrolled growth and spread of abnormal cells that can invade surrounding tissues and metastasize to distant organs (1, 30). The most common cancer types worldwide include breast, lung, colorectal, prostate, and stomach cancers (2). Risk factors include aging, genetic predisposition, tobacco and alcohol use, unhealthy diet, physical inactivity, environmental carcinogen exposure, and chronic infections such as hepatitis and human papillomavirus (3).

According to the World Health Organization (WHO), cancer was the second leading cause of death globally in 2020, accounting for nearly 10 million deaths. In the United Arab Emirates (UAE), cancer is the third leading cause of death, following cardiovascular diseases and accidents (4). Data from the UAE National Cancer Registry reported 1,193 new cancer cases among citizens in 2019, with malignant tumors accounting for 93% of cases. Breast, thyroid, colorectal, leukemia, and lung cancers are the most prevalent types in the country (4). The growing burden of cancer in the UAE has been linked to modifiable risk factors such as obesity, sedentary lifestyle, and smoking.

Early detection through routine screening plays a critical role in reducing cancer-related mortality by enabling timely diagnosis and treatment. Established screening modalities, such as mammograms for breast cancer, Pap smears for cervical cancer, and colonoscopies for colorectal cancer, have been shown to improve survival and reduce treatment costs (5). Despite the availability of screening services, participation remains inconsistent.

A cross-sectional survey in the UAE revealed that 91.8% of the importance of early cancer detection, however, screening uptake was considerably lower: 22.5% reported undergoing mammography, 12.8% had completed Pap smears, and 6.6% had participated in colorectal cancer screening (6). Another study showed that 34% of women over the age of 40 at Sheikh Shakhbout Medical City had never scheduled a mammogram, with higher rates (46%) reported in Sharjah (6). The findings highlight a gap between awareness and actual screening behavior.

In the UAE, sociocultural norms may influence attitudes toward preventive healthcare, particularly among women, where screening can sometimes be perceived as uncomfortable or socially sensitive (7). The country’s diverse population, including a substantial expatriate community, may also experience variability in healthcare and utilization (6). Additional barriers such as fear of diagnosis concerns about screening procedures, and limited familiarity with available services have been reported (8).

Although previous studies have examined screening behaviors in the UAE, most have focused on specific cancer type or limited population groups, limiting generalizability. For example, a recent study examined awareness of skin cancer screening but lacked broad geographic presentation (9). Comprehensive evidence simultaneously assessing cancer-related knowledge, attitudes, perceived motivators and barriers, and sociodemographic predictors of screening participation across cancer types in the general adult population remains limited. Therefore, this study aimed to assess cancer-related knowledge, attitudes, perceived motivators and barriers, and factors associated with lifetime (“ever”) cancer screening participation among adults in the UAE.

## 2 Materials and methods

### 2.1 Study design and subjects

This cross-sectional study was conducted between October 2024 and May 2025 across the UAE. A self-administered online questionnaire, available in both English and Arabic, was distributed using a convenience sampling approach through widely used digital platforms, including WhatsApp, Facebook, and Twitter. Eligibility criteria included UAE residency and a minimum age of 18 years. Before proceeding with the questionnaire, participants were required to indicate informed consent by checking a designated box. The survey was anonymous, and no personal identifiable or culturally sensitive information was collected. To enhance presentation, demographic response patterns were periodically reviewed, and outreach efforts were adjusted to mitigate potential sampling bias. The average completion time for the survey was approximately 10 min.

### 2.2 Study instrument

The survey was developed based on a comprehensive review of relevant literature (10, 11). It began with a short introduction describing the study objectives and emphasizing confidentiality and anonymity. The survey consisted of five sections. The first section collected socio-demographic data, including age, gender, marital status, education level, area of residence, monthly income, employment sector, health insurance status, and family history of cancer. It also included questions on lifestyle-risk factors such as tobacco and alcohol use, diet, and physical activity. These items were used to construct the cancer risk factor exposure score as an unweighted additive index (range 0–13) by summing prespecified item scores: smoking status (0 = non-smoker, 1 = former smoker, 2 = current smoker), physical inactivity (1 = not exercising for at least 30 min three times per week), low fruit and vegetable intake (1 = not consuming more than five servings per day), regular red/processed meat intake (1 = yes), high-sugar food intake (1 = yes), alcohol consumption (1 = yes), excessive sun exposure (0–4 from never to every day), family history of cancer (1 = yes), and overweight/obesity (1 = overweight/obese vs. underweight/normal). Screening behavior was assessed using the following item: “Have you ever undergone any cancer screening test (e.g., mammography, Pap smear, colonoscopy, fecal occult blood test, or other cancer screening test)?” Response options were “Yes” or “No.” Responses were dichotomized (Yes = 1, No = 0) and used as the dependent variable in the regression analysis. This measure reflects lifetime (“ever”) screening and does not assess adherence to age-, sex-, or interval-specific national screening recommendations or whether tests were conducted for preventive screening versus diagnostic evaluation. The second section comprised of 59 items assessing general knowledge, screenable cancer types, warning signs and symptoms, and environmental risk factors. Responses were recorded as “Yes,” “No,” or “Not Sure. A composite knowledge score was calculated, with a maximum possible score of 59. The third part assessed attitudes toward cancer and screening using a 5-point Likert scale ranging from “Strongly disagree” to “Strongly agree.” The next part asked participants about factors that would encourage them to undergo cancer screening. The last 12-item part explored the perceived barriers to screening.

The survey underwent forward and backward translation to ensure linguistic accuracy in both English and Arabic. A multidisciplinary expert panel consisting of a family physician, oncologist, and public health academic evaluated content validity via ensuring relevancy and comprehensiveness of the questionnaire. Based on the feedback received, minor adjustments were made, including re-wording a few items to enhance clarity, resolving ambiguities, and refining the layout for better flow and ease of completion. A pilot test with 20 individuals was conducted to assess clarity and appropriateness of the questionnaire. The piloted individuals were excluded from the main study. The internal consistency of the knowledge (Cronbach’s alpha = 0.91) and attitude (Cronbach’s alpha = 0.90) sections demonstrated the reliability of the study instrument. The online questionnaire was programmed to require responses to all study variables before submission; therefore, no missing data were present in the final dataset.

### 2.3 Sample size calculations

To calculate the minimum required sample size, the following equation was used: (12).


$$n=\frac{z^{2}p\;(1-p)}{d^{2}}$$

where *p* represents the predicted percentage (assumed to be 0.5 for maximum variability), *d* is the margin of error (0.05), and confidence level is 95% (*z* = 1.96). Based on these parameters, a sample size of 385 was determined.

### 2.4 Ethical approval

Ethical approval for this study was granted by the research ethics committee at Al Ain University – Abu-Dhabi Campus (Ref #: COP/AREC/AD/07). All participants were informed about the study’s purpose, and digital informed consent was obtained prior to participation.

### 2.5 Statistical analysis

The Statistical Package for the Social Sciences (SPSS, version 28, Illinois, New York, United States) was used to perform the data analysis. Descriptive statistics were used to summarize the data, medians and interquartile range (IQR) were used for continuous variables, while frequencies and percentages to express categorical variables. Binary logistic regression was used to examine factors associated with cancer screening practice (ever screened: yes/no). Univariate logistic regression analyses were first performed to estimate crude odds ratios (ORs) with 95% confidence intervals (CIs) for each independent variable. Independent variables were selected *a priori* for inclusion in the multivariable model based on theoretical relevance and evidence from previous literature on determinants of cancer screening behaviors. A multivariable binary logistic regression model was then fitted using a forced-entry (Enter) approach including age, sex, marital status, educational level, monthly income, health insurance status, knowledge score, attitude score, and the cancer risk factor exposure score, and adjusted odds ratios (AORs) with 95% CIs were reported. Knowledge, attitude, and risk factor exposure scores were retained as continuous variables in regression models. Multicollinearity was assessed using the variance inflation factor (VIF), with values <3.0 considered acceptable, and statistical significance was set at *p* < 0.05.

## 3 Results

A total of 811 individuals participated in the study, of whom 54.1% were female, with a median age (IQR) of 27 (21–38) years (Table 1). Most participants had less than a university degree (54.0%), lived in urban areas (79.9%), reported a monthly income below10.000 AED (59.9%), had not worked in the medical field (68.2%), had health insurance (72.5%), and had never performed cancer screening (63.5%). Regarding lifestyle factors, most participants were not smokers (47.5%) and did not drink alcohol (70.4%). The majority did not regularly consume processed meat regularly (66.7%) and did not consume several servings of fruits and vegetables daily (67.0%). More than half did not exercise for at least 30 min regularly (54.1%). Most participants had no family history of cancer (71.9%), and 24.0% were classified as obese. The median cancer risk factor exposure score was 5 (3–6) out of a maximum of 13. Overall, 36.5% of participants reported having ever undergone any form of cancer screening based on self-reported lifetime participation. The median knowledge score was 28 (21–34) out of 59, indicating a room for improvement across general cancer knowledge, screenable cancer types, warning signs and symptoms, and environmental risk factors. Awareness was highest for breast cancer (71.4%) and comparatively lower for colorectal (49.8%) and lung cancer (46.0%) (Table 2). Recognition of warning symptoms varied, with unexplained swelling or lump most frequently identified (66.2%) and persistent difficulty swallowing least recognized (43.0%) (Table 3). For environmental risk factors, smoking and alcohol were most frequently identified (68.8%), whereas breastfeeding for less than 6 months was less commonly recognized (37.9%) (Table 3).

**Table 1:** Sociodemographic characteristics of the participants (*n* = 811).

Characteristics		Frequency (%) or median (IQR)
Age (years)		27 (21–38)
Sex	Female	439 (54.1%)
	Male	372 (45.9%)
Marital status	Other	418 (51.5%)
	Married	393 (48.5%)
Educational level	Less than university degree	438 (54.0%)
	University degree	373 (46.0%)
Residential area	Rural	163 (20.1%)
	Urban	648 (79.9%)
Monthly income	≤10,000 AED	486 (59.9%)
	>10,000 AED	325 (40.1%)
Medical field	No	553 (68.2%)
	Yes	258 (31.8%)
Do you have health insurance?	No	223 (27.5%)
	Yes	588 (72.5%)
Performing cancer screening	No	515 (63.5%)
	Yes	296 (36.5%)
Source of health or disease information, for example, cancer	Books/magazines	55 (6.8%)
	Friends/relatives	62 (7.6%)
	Healthcare staff	136 (16.8%)
	Internet and social media	263 (32.4%)
	Schools/university	165 (20.3%)
	TV	130 (16.0%)
Risk factors for cancer		
Smoking	Non-smoker	385 (47.5%)
	Former smoker	311 (38.3%)
	Current smoker	115 (14.2%)
Exercise for at least 30 min	Yes	372 (45.9%)
	No	439 (54.1%)
Eat more than five fruits and vegetables per day	Yes	268 (33.0%)
	No	543 (67.0%)
Eat red or processed meat on a regular basis	No	541 (66.7%)
	Yes	270 (33.3%)
Eat high-sugar food	No	527 (65.0%)
	Yes	284 (35.0%)
Drink alcohol	No	571 (70.4%)
	Yes	240 (29.6%)
Get exposed excessively to sunlight	Never	43 (5.3%)
	Rarely	242 (29.8%)
	Sometimes	288 (35.5%)
	Most of the time	169 (20.8%)
	Everyday	69 (8.5%)
Family history of cancer	No	583 (71.9%)
	Yes	228 (28.1%)
Obese	No	616 (76.0%)
	Yes	195 (24.0%)
Median risk score	>5	357 (44.0%)
	≤ 5	454 (56.0%)

**Table 2:** General knowledge and awareness of the types of cancer to be screened for (*n* = 811).

General knowledge	No	I do not know	Yes
Cancer is a fatal disease and can lead to death.	157 (19.4%)	87 (10.7%)	567 (69.9%)
The causes of cancer might be genetic and or environmental factors.	279 (34.4%)	141 (17.4%)	391 (48.2%)
Obesity is a known risk factor for various cancers, including breast, colon, and kidney cancer.	245 (30.2%)	252 (31.1%)	314 (38.7%)
Not all tumors are cancerous; benign tumors do not spread to other body parts.	243 (30.0%)	199 (24.5%)	369 (45.5%)
Cancer is a preventable condition.	260 (32.1%)	182 (22.4%)	369 (45.5%)
Protection against cancer starts in childhood.	247 (30.5%)	198 (24.4%)	366 (45.1%)
The self-examination of individuals is vital to notice signs of cancer.	249 (30.7%)	172 (21.2%)	390 (48.1%)
Recovery rates increase when cancer is detected in the early stages.	225 (27.7%)	152 (18.7%)	434 (53.5%)
Treatment options depend on the type and stage of cancer.	231 (28.5%)	156 (19.2%)	424 (52.3%)
Vaccinations, such as the HPV vaccine, can prevent certain cancers.	215 (26.5%)	231 (28.5%)	365 (45.0%)
Types of cancer that could be screened for			
Breast cancer	118 (14.5%)	114 (14.1%)	579 (71.4%)
Colon cancer	204 (25.2%)	203 (25.0%)	404 (49.8%)
Anal cancer^*^	228 (28.1%)	237 (29.2%)	346 (42.7%)
Prostate cancer	270 (33.3%)	200 (24.7%)	341 (42.0%)
Lung cancer	247 (30.5%)	191 (23.6%)	373 (46.0%)
Ovarian cancer^*^	249 (30.7%)	218 (26.9%)	344 (42.4%)
Blood cancer^*^	253 (31.2%)	199 (24.5%)	359 (44.3%)
Lymph node cancer^*^	238 (29.3%)	231 (28.5%)	342 (42.2%)
Brain cancer^*^	245 (30.2%)	219 (27.0%)	347 (42.8%)
Bone cancer^*^	255 (31.4%)	196 (24.2%)	360 (44.4%)

* The correct answer is “no”.

**Table 3:** Knowledge about warning symptoms and environmental risk factors (*n* = 811).

	No	I do not know	Yes
Warning symptoms of cancer			
Unexplained swelling lump	158 (19.5%)	116 (14.3%)	537 (66.2%)
Unexplained pain	194 (23.9%)	162 (20.0%)	455 (56.1%)
Unexplained bleeding	204 (25.2%)	167 (20.6%)	440 (54.3%)
Persistent cough hoarseness	224 (27.6%)	198 (24.4%)	389 (48.0%)
Persistent change in bowel habit	226 (27.9%)	212 (26.1%)	373 (46.0%)
Persistent difficulty swallowing	253 (31.2%)	209 (25.8%)	349 (43.0%)
Nonhealing ulcer	199 (24.5%)	197 (24.3%)	415 (51.2%)
Unexplained weight loss	222 (27.4%)	177 (21.8%)	412 (50.8%)
Fatigue or extreme tiredness	212 (26.1%)	171 (21.1%)	428 (52.8%)
Unexplained pain in the bladder when passing urine	220 (27.1%)	195 (24.0%)	396 (48.8%)
Fever or night sweats	239 (29.5%)	207 (25.5%)	365 (45.0%)
Headaches	243 (30.0%)	183 (22.6%)	385 (47.5%)
Vision or hearing problems	245 (30.2%)	203 (25.0%)	363 (44.8%)
Environmental risk factors for cancer			
Infections and parasites	287 (35.4%)	154 (19.0%)	370 (45.6%)
Lifestyle includes smoking and alcohol consumption	167 (20.6%)	86 (10.6%)	558 (68.8%)
Exposure to UV light and tanning devices	268 (33.0%)	157 (19.4%)	386 (47.6%)
Using hormone replacement therapy	270 (33.3%)	201 (24.8%)	340 (41.9%)
Exposure to harmful substances or agents in the workplace.	253 (31.2%)	178 (21.9%)	380 (46.9%)
Indoor combustion	257 (31.7%)	235 (29.0%)	319 (39.3%)
Exposure to soot and wood dust	274 (33.8%)	201 (24.8%)	336 (41.4%)
Environmental and industrial pollution	243 (30.0%)	196 (24.2%)	372 (45.9%)
Exposure to nuclear rays and rays	243 (30.0%)	172 (21.2%)	396 (48.8%)
Having multiple sex partners	245 (30.2%)	209 (25.8%)	357 (44.0%)
Processed food	241 (29.7%)	187 (23.1%)	383 (47.2%)
Long-term use of preservatives	250 (30.8%)	164 (20.2%)	397 (49.0%)
High sugar diet	269 (33.2%)	178 (21.9%)	364 (44.9%)
Following low fiber diet	255 (31.4%)	195 (24.0%)	361 (44.5%)
Consuming red meat	287 (35.4%)	171 (21.1%)	353 (43.5%)
Not cleaning the water pipe from which you smoke	241 (29.7%)	213 (26.3%)	357 (44.0%)
Breastfeeding for less than 6 months	267 (32.9%)	237 (29.2%)	307 (37.9%)
Uranium exposure	245 (30.2%)	170 (21.0%)	396 (48.8%)
Exposure to paints	246 (30.3%)	206 (25.4%)	359 (44.3%)
Tobacco exposure	251 (30.9%)	179 (22.1%)	381 (47.0%)
Diesel exhaust	236 (29.1%)	200 (24.7%)	375 (46.2%)
Plastic containers	229 (28.2%)	202 (24.9%)	380 (46.9%)
Household cleaning products	237 (29.2%)	215 (26.5%)	359 (44.3%)
Pesticides	238 (29.3%)	197 (24.3%)	376 (46.4%)
Naphthalene	234 (28.9%)	220 (27.1%)	357 (44.0%)
Asbestos	243 (30.0%)	197 (24.3%)	371 (45.7%)
Median knowledge score	>28		384 (47.3%)
	**≤**28		427 (52.7%)

The median attitude score was 25 (13–20) out of 35, reflecting a moderately positive but inconsistent attitude toward cancer and screening. Agreement was lowest for the statement that “Self-checks for cancer help to detect the early signs of the cancer” (45.9%) (Table 4).

**Table 4:** Attitudes towards cancer and cancer screening (*n* = 811).

Attitude	Strongly disagree	Disagree	Neutral	Agree	Strongly agree
It is important for me to know about cancer.	59 (7.3%)	73 (9.0%)	286 (35.3%)	251 (30.9%)	142 (17.5%)
Cancer screening should be implemented on a large scale.	58 (7.2%)	93 (11.5%)	265 (32.7%)	233 (28.7%)	162 (20.0%)
I believe that cancer screening is effective to help in the early detection of cancer.	79 (9.7%)	105 (12.9%)	212 (26.1%)	242 (29.8%)	173 (21.3%)
Cancer is diagnosed at an early stage; the treatment outcomes can be better.	69 (8.5%)	99 (12.2%)	267 (32.9%)	216 (26.6%)	160 (19.7%)
Physical clinical examination by the physician helps detect the early stages of cancer.	63 (7.8%)	96 (11.8%)	262 (32.3%)	235 (29.0%)	155 (19.1%)
Self-checks for cancer help to detect the early signs of the cancer.	65 (8.0%)	106 (13.1%)	267 (32.9%)	225 (27.7%)	148 (18.2%)
Awareness about cancer is important to reduce risk.	63 (7.8%)	103 (12.7%)	243 (30.0%)	231 (28.5%)	171 (21.1%)
Median attitude score [frequency (%)]	>25	351(43.3%)			
	**≤**25	460 (56.7%)			

**Figure 1:**
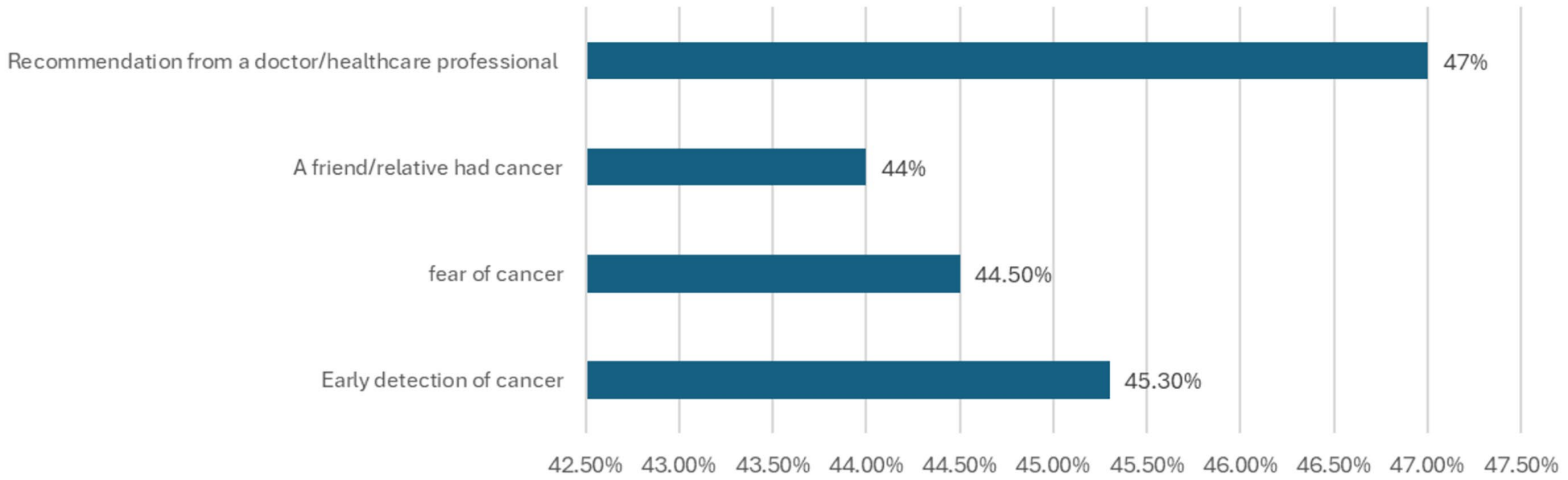
Factors that encourage participants to undergo cancer screening.

**Figure 2:**
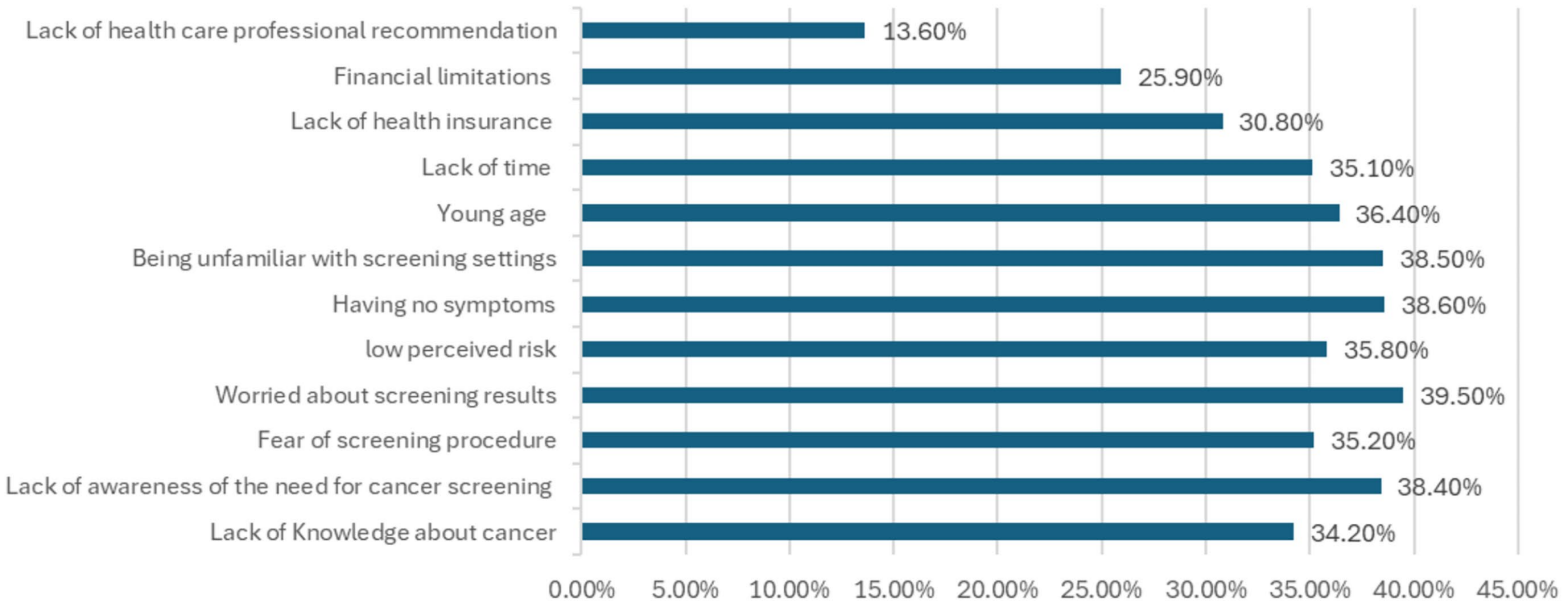
Barriers to cancer screening.

As shown in Figure 1, physician recommendation (47.0%) and the belief in early detection (45.3%) were the most commonly reported motivators for screening. Reported barriers (Figure 2) included fear of screening results (39.5%), absence of symptoms (38.6%), and unfamiliarity with screening locations (38.5%). Lack of health care professional recommendation was the least frequently reported barrier (13.6%).

Logistic regression analysis (Table 5) demonstrated that higher knowledge (OR = 1.022; 95%CI = 1.007–1.037; *p* = 0.005) and more positive attitudes (OR = 1.091; 95%CI = 1.062–1.120; *p* < 0.001) were significantly associated with higher odds screening participation. In contrast, increasing age (OR = 0.970; 95%CI = 0.955–0.985; p < 0.001), and lower monthly income (OR = 0.689, 95%CI = 0.483–0.982; *p* = 0.04) were associated with decreased odds of performing cancer screening.

**Table 5:** Variables associated with performing cancer screening.

		Crude OR	Adjusted OR	95% confidence interval		*p*-value
				Lower	Upper	
Age (years)		0.98 (0.96–0.99)	0.970	0.955	0.985	<0.001
Knowledge/awareness score		1.02 (1.00–1.03)	1.022	1.007	1.037	0.005
Attitude score		1.08 (1.06–1.11)	1.091	1.062	1.120	<0.001
Medical field		1.36 (0.80–2.31)	0.925	0.514	1.665	0.796
Gender	Female vs. male	1.34 (1.00–1.79)	1.267	0.932	1.724	0.131
Marital status	Single vs. married	1.12 (0.84–1.49)	0.827	0.586	1.166	0.278
Educational level	Less than university vs. university degree	1.09 (0.82–1.456)	1.294	0.946	1.770	0.107
Monthly income	≤10,000 AED vs. >10,000 AED	1.10 (0.82–1.48)	0.689	0.483	0.982	0.040
Do you have health insurance	No vs. yes	1.04 (0.75–1.43)	1.214	0.858	1.719	0.274
Risk score of developing cancer		1.00 (0.92–1.08)	0.997	0.915	1.087	0.948

## 4 Discussion

The findings of this study provide insightful understanding of the UAE public knowledge, attitude, and perceived barriers to cancer screening. Overall, the participants had moderate cancer knowledge, ambivalent attitudes, and substantial, yet modifiable, barriers to screening. These findings are consistent with recent evidence from the country and the wider Gulf region (21, 22).

In this study, recognition of basic concepts, such as cancer can be fatal and that early detection improves outcomes, was common, but knowledge about specific screenable cancers and environmental risks was uneven. This aligned with a 2023 population-based UAE survey reported high awareness of the idea of “early detection,” yet variable understanding of which cancers are screenable (6). Notably, awareness of the human papillomavirus (HPV) vaccine as a means of cancer prevention was relatively low among participants (45.0%), underscoring a critical knowledge gap that warrants attention. These findings highlight the importance of implementing targeted public health strategies, such as community-based awareness initiatives, school-level education programs, and proactive counseling by healthcare providers, to enhance public understanding of HPV vaccination as an essential tool in cancer prevention.

Awareness among the population of the study was highest for breast cancer and comparatively lower for colorectal and lung cancers, likely reflecting the stronger visibility of breast cancer awareness campaigns nationally. Women aged 40–69 are typically targeted by screening recommendations, yet engagement in preventive behaviors has been reported as low in prior studies (23). A 2025 community study reported a median colorectal cancer (CRC) knowledge score of 10/22, with only 29% aware of the national screening program and 10.1% screening uptake among eligible adults (24). These patterns suggest that awareness alone is insufficient and that system navigation and risk perception remain key barriers to screening engagement (31). Similar gaps have been observed in Sweden and Denmark, where public understanding of modifiable risk factors like obesity, alcohol, and processed meats is low (25). In Saudi Arabia’s Jazan region, limited awareness of smoking as a key lung cancer risk factor further highlights the need for targeted education (26).

Although participants generally held favorable attitudes toward cancer screening, confidence in self-examination as an effective early detection tool was notably low, the least supported belief in our survey. Additionally, fewer than half endorsed comprehensive screening programs, pointing to a gap between positive perceptions and actual preventive behavior. Similar trends have been observed globally. Studies among women in Jeddah, Saudi Arabia (13) and female university students in Cameroon (14) found moderately positive attitudes toward early detection and self-examination, yet limited participation in screening. In Kerala, India, while the general population supported large-scale screening initiatives in principle, this did not consistently translate into action (15). Together, these findings illustrate a persistent attitude–behavior gap in cancer screening participation. To improve cancer screening uptake, efforts must go beyond raising awareness and focus on strengthening confidence in screening methods, promoting early detection as a norm, and ensuring services are accessible, culturally appropriate, and easy to navigate.

Despite generally favorable knowledge and attitudes toward cancer screening, 36.5% of participants reported having undergone a cancer screening test at least once in their lifetime. Considering the relatively young age distribution of the sample (median age 27 years), this prevalence may reflect opportunistic screening, diagnostic investigations performed outside organized screening programs, or differences in how participants interpreted the concept of cancer screening. This aligns with regional evidence indicating that awareness alone does not guarantee preventive action, as consistently low screening participation has been observed across studies despite reasonable knowledge levels (16, 22). In our study, this gap highlights that even when participants recognize the importance of early detection, such awareness does not always lead to screening behavior. Accordingly, actual participation serves as an important measure for understanding preventive practices and identifying factors that determine whether knowledge is converted into action. These findings underscore the importance of analyzing screening predictors to identify barriers and facilitators that guide targeted interventions and translate awareness into preventive action.

The interpretation of screening uptake should be in light of the age distribution of the study sample. The median participants age was 27 years, which is younger than the age groups targeted by organized cancer screening programs in the UAE, particularly for breast and colorectal cancers (40–75 years). Screening uptake was assessed as lifetime (“ever”) participation and was not evaluated according to age-, sex-, or interval-specific eligibility criteria. Consequently, a substantial proportion of participants were not yet eligible for routine screening, which may partly explain the observed screening prevalence. Therefore, the reported uptake should not be interpreted as adherence to national screening recommendations. However, the inclusion of adults aged 18 years and older was intentional, as the study aimed to assess cancer-related knowledge, attitudes, and perceived barriers within the general adult population. Understanding awareness and perceptions among younger adults is important, as these factors may influence future engagement with screening when individuals become age-eligible.

The current study revealed that obtaining higher knowledge scores substantially improved the odds of utilizing cancer screening services. Similarly, more favorable attitudes toward cancer screening increased the likelihood of being screened. Across multiple settings, individuals with higher knowledge levels, stronger intentions, and positive attitudes were more likely to undergo screening. For example, studies based on the Theory of Planned Behavior in Iran (17) and meta-analytic findings from Sub-Saharan Africa (18) consistently demonstrated that awareness, perceived control, and social norms significantly influenced screening behaviors. Similarly, awareness and favorable attitudes were associated with higher screening rates, particularly among women in the UAE (6).

In our multivariate analysis, older age was found to be significantly related to lower odds of reporting lifetime cancer screening participation. This result is surprising, given that screening program eligibility criteria usually target older individuals. However, the relatively young age distribution in our sample (median age 27 years) and the convenience sampling method may have contributed to this finding. It is also possible that our sample members, particularly the younger ones, were more health-conscious or more likely to respond to health promotion messages, whereas older age groups were underrepresented. As such, this age effect should be interpreted cautiously and not generalized beyond this sample.

In our multivariate model, increasing age was associated with lower odds of reporting lifetime cancer screening participation. This finding contrasts with screening eligibility guidelines, which typically target older adults. However, the relatively young age distribution of our sample (median 27 years) and the convenience sampling approach may have influenced this association. It is possible that younger respondents in our sample were more health-conscious or more responsive to preventive health messaging, while older age groups were underrepresented. Therefore, this observed age effect should be interpreted cautiously and not generalized to the broader UAE population.

The main barriers identified in our study, including fear of results, absence of symptoms, and lack of awareness of screening locations, align closely with findings from recent UAE and regional studies. For instance, 70.0% of participants in a previous UAE survey cited fear of test results, 68.5% discomfort, 68.0% perceived pain, and 71.9% reported not being offered screening by a physician as reasons for non-participation (22). Similarly, in Qatar, 60.6% of unscreened adults believed they were not at risk due to lack of symptoms (20). A Saudi study echoed these findings, reporting fear of results (28.9%), shyness (26.5%), fear of pain (20.6%), and absence of symptoms as common deterrents (27). These patterns are consistent with broader regional findings. Interestingly, our participants reported “lack of physician recommendation” less frequently than other barriers, which contrasts with many Gulf studies where provider advice is a key driver of screening uptake. In the 2023 UAE CRC study, physician recommendation was the most cited reason for getting screened (22). This discrepancy may be due to the younger, urban demographic in our sample, many of whom are not yet eligible for screening, and a greater reliance on internet-based health information. However, as noted in a systematic review, addressing “no symptom” misconceptions, improving system navigation, and increasing opportunistic clinician invitations are crucial to converting awareness into action (28).

Finally, low rates of regular physical activity and limited fruit and vegetable intake among participants mirror regional patterns, where modifiable risk factors remain widespread despite increasing awareness. While knowledge is positively associated with screening behavior in UAE CRC studies, a 2024 MENA scoping review emphasizes that smoking, poor diet, and inactivity continue to fuel the cancer burden (29). Importantly, perceived benefits and barriers appear to influence screening behavior more strongly than knowledge alone. A 2024 meta-analysis found that individuals who were screened had significantly lower perceived barrier scores (SMD − 0.466) and higher benefit scores (SMD 0.379) compared to those who were not (28).

From a policy standpoint, these results underscore the necessity of enhancing opportunistic screening invites in primary care settings, raising public knowledge of eligibility requirements, and dispelling myths like the idea that a lack of symptoms indicates low risk. Improving screening service navigation and including screening reminders into regular medical visits could aid in putting awareness into practice within the UAE healthcare system.

### 4.1 Study limitations

Given the cross-sectional design and convenience sampling approach, these results should be regarded with caution, as they reflect associations rather than causal relationships and may be subject to selection bias. The use of a self-report instrument could increase the risk of social-desirability bias. Furthermore, while the study’s substantial sample size enhances the generalizability of its findings, it should be noted that the socio-demographic characteristics of the current study participants may not fully reflect the broader population in the UAE. Moreover, the screening behavior was assessed as lifetime self-reported participation and was not evaluated according to age-, sex-, or interval-specific national guidelines. Given the relatively young median age of participants (27 years), many were below the recommended age for routine screening. Therefore, these findings should not be interpreted as reflecting adherence to national screening recommendations. Future studies should focus on age-eligible populations and consider probability-based sampling methods to improve generalizability. Despite these limitations, the study addresses an important public health issue in the UAE context, includes a relatively large sample size, utilizes a bilingual instrument with good internal consistency, and provides actionable findings regarding motivators and barriers to cancer screening.

## 5 Conclusion

Among the participants in this study, cancer knowledge was moderate, attitudes were mixed, and lifetime screening participation was modest, with fear, low perceived risk, and navigation gaps emerging as key barriers. These findings highlight the need for culturally appropriate educational strategies targeting these barriers. Cancer education should be integrated into community outreach programs, workplace health initiatives, and school curricula, while primary healthcare providers, including family doctors and pharmacists, should offer education during routine consultations. Addressing these gaps through culturally sensitive education and improved healthcare system navigation may enhance future screening participation and facilitate earlier detection.

## Data Availability

The raw data supporting the conclusions of this article will be made available by the authors, without undue reservation.
